# T cell exhaustion landscapes and therapeutic modulation in cancer immunity

**DOI:** 10.3389/fcell.2026.1827716

**Published:** 2026-05-14

**Authors:** Jhommara Bautista, Andrés López-Cortés

**Affiliations:** Cancer Research Group (CRG), Faculty of Medicine, Universidad de Las Américas, Quito, Ecuador

**Keywords:** cancer immunotherapy, immune checkpoint blockade, progenitor exhausted T cells, t cell exhaustion, tumor microenvironment

## Abstract

T cell exhaustion is a central framework for explaining why antitumor T cell responses often fail despite persistent antigen exposure and immune infiltration. Rather than a single dysfunctional endpoint, exhaustion is increasingly understood as a structured and dynamic continuum of antigen-experienced CD8^+^ T cell states that differ in proliferative capacity, effector potential, epigenetic constraint, metabolic fitness, and spatial distribution within tumors. This view has major therapeutic implications because clinically relevant interventions can remodel exhausted-state composition and function without fully restoring a non-exhausted identity. In this review, we examine the organization of exhausted T cell states from progenitor-like to terminal compartments and discuss how TOX-linked survival programs, epigenetic fixation, and tumor-imposed metabolic and spatial constraints stabilize exhausted fate under chronic stimulation. We highlight the role of progenitor exhausted T cells in sustaining therapeutic responsiveness, explain why reinvigoration after checkpoint blockade is often partial rather than transformative, and evaluate emerging strategies to modulate exhaustion dynamics, including combination immunotherapy and engineered control systems in CAR T cells. Together, these concepts support a shift from viewing exhaustion as a binary defect to understanding it as a constrained state system that can be measured, preserved, and selectively redirected. Defining which exhausted states remain productively controllable, and under what conditions, will be essential for developing more durable and mechanistically informed cancer immunotherapies.

## Introduction

T cell exhaustion has become a central framework for explaining why antitumor T cell responses often fail to maintain effective control despite persistent antigen exposure and immune infiltration (Zajac et al., 1998; Wherry, 2011). In many tumors, antigen-experienced CD8^+^ T cells persist but adopt phenotypic and transcriptional programs characterized by inhibitory receptor expression, altered effector function, and limited proliferative capacity (Guo et al., 2025; Wu et al., 2026). These features, however, are not uniform. Their extent and combinations vary across tumor types, tissue compartments, disease stages, and treatment contexts, indicating that exhaustion is best interpreted as a heterogeneous and context-dependent state rather than a single fixed phenotype (Blank et al., 2019). This concept has become especially important in modern cancer therapy because both immune checkpoint blockade and adoptive cellular immunotherapies operate within, and are constrained by, the differentiation state of tumor-reactive T cells. Immune checkpoints such as programmed cell death protein 1 (PD-1) and cytotoxic T-lymphocyte-associated protein 4 (CTLA-4) normally restrain T cell activation to preserve immune homeostasis, but in cancer these pathways can also suppress effective antitumor responses (Bautista et al., 2026; Miller et al., 2019; Siddiqui et al., 2019; Leach et al., 1996). Similarly, chimeric antigen receptor T (CAR-T) cells can mediate potent cytotoxicity yet remain vulnerable to chronic antigen exposure, tonic signaling, and suppressive microenvironments that promote dysfunctional or exhaustion-like states (Kyriakidis et al., 2025; Long et al., 2015; Gomes-Silva et al., 2017). Thus, T cell exhaustion is not only a descriptive framework for chronic immune dysfunction, but also a clinically important determinant of therapeutic responsiveness and durability.

Recent high-dimensional studies have reinforced this view by resolving multiple exhausted CD8^+^ T cell subsets, including less differentiated progenitor-like populations, more differentiated terminal populations, and intermediate states that connect them (Beltra et al., 2023; Beltra et al., 2020). Importantly, the continuum of exhausted CD8^+^ T cell states has been delineated most rigorously in murine models of chronic antigen exposure, in which progenitor, intermediate, and terminally differentiated Tex subsets and their developmental relationships have been resolved with greater mechanistic precision (Beltra et al., 2020; Pritykin et al., 2021). In human tumors, analogous progenitor-like and more differentiated exhausted populations have been described, including TCF1^+^PD-1^+^ stem-like subsets and tumor-reactive exhausted CD8^+^ T cells, but their alignment with murine Tex states is not strictly one-to-one (Siddiqui et al., 2019; Oliveira et al., 2021). Rather, human exhausted T cell states appear to be more variably shaped by tumor context, tissue residency programs, patient-to-patient heterogeneity, and prior or ongoing therapy, such that phenotypic boundaries and inferred developmental trajectories are often less uniform than in murine systems (Barsch et al., 2022; Caushi et al., 2021). This framework is useful because it shifts the focus from assigning rigid labels to understanding how distinct exhausted states are distributed and how they change under chronic antigen stimulation and therapy. It also provides a more precise basis for interpreting therapeutic responses, since interventions may alter the composition, function, and spatial distribution of exhausted T cell populations without necessarily restoring a fully non-exhausted identity (Pauken et al., 2016; Philip et al., 2017).

This distinction is particularly important in cancer immunotherapy. Immune checkpoint blockade can enhance proliferation and selected effector functions within exhausted compartments, yet clinical responses remain heterogeneous and are often limited in durability (Huang et al., 2017; Miller et al., 2019). Such outcomes suggest that reinvigoration is frequently partial and shaped by multiple constraints, including stable regulatory programs, the availability of progenitor-like exhausted reservoirs, and microenvironmental pressures such as metabolic stress, suppressive ligands, and spatially organized inhibitory niches (Scharping et al., 2016; Schürch et al., 2020; Bautista et al., 2025a). Accordingly, reinvigoration is most appropriately viewed as a measurable shift in function and state composition under perturbation, rather than as evidence of complete identity reversal (Yost et al., 2019; Jansen et al., 2019). Likewise, CAR-T cells can undergo exhaustion-like remodeling under persistent antigen exposure and tonic signaling, making exhaustion biology relevant not only to endogenous antitumor immunity but also to engineered cell therapies (Selli et al., 2023; Long et al., 2015).

In this review, we examine T cell exhaustion as a structured and dynamic state system with direct relevance to cancer therapy. We discuss the organization of exhausted T cell states, the survival-linked regulatory programs that stabilize them, the epigenetic constraints that can limit reinvigoration, the role of progenitor-like exhausted subsets in sustaining therapeutic responses, and the bioenergetic and spatial factors that shape state persistence within tumors. We also review emerging strategies to therapeutically modulate exhaustion dynamics, including combination immunotherapy and engineered control circuits in CAR T cell systems. Throughout, we emphasize mechanistically grounded and experimentally testable interpretations that distinguish transient functional improvement from durable changes in exhausted-state organization.

## T cell exhaustion as a dynamic state space

T cell exhaustion is most informative when viewed as a continuous and heterogeneous state space rather than a binary failure mode. In chronic infection and cancer, antigen-experienced CD8^+^ T cells occupy multiple partially overlapping transcriptional and phenotypic configurations, and high-dimensional profiling consistently reveals structured heterogeneity within the exhausted compartment (Beltra et al., 2020; Andreatta et al., 2021). This framework is useful because it captures exhaustion as a set of related cellular states defined by molecular similarity, while avoiding the assumption that each cluster represents a fixed lineage or discrete cell type (Yost et al., 2019; Satpathy et al., 2019; Blank et al., 2016). At the same time, the developmental organization of exhausted CD8^+^ T cell states has been established most rigorously in murine systems under chronic antigen exposure, where progenitor-like, intermediate, and terminally differentiated subsets and their hierarchical relationships have been resolved with substantial mechanistic precision. In human tumors, analogous states have been identified, but they are more often inferred from cross-sectional single-cell, clonotypic, and phenotypic analyses than resolved with the same degree of developmental certainty (Beltra et al., 2020; Miller et al., 2019; Utzschneider et al., 2016; Yost et al., 2019).

**FIGURE 1:**
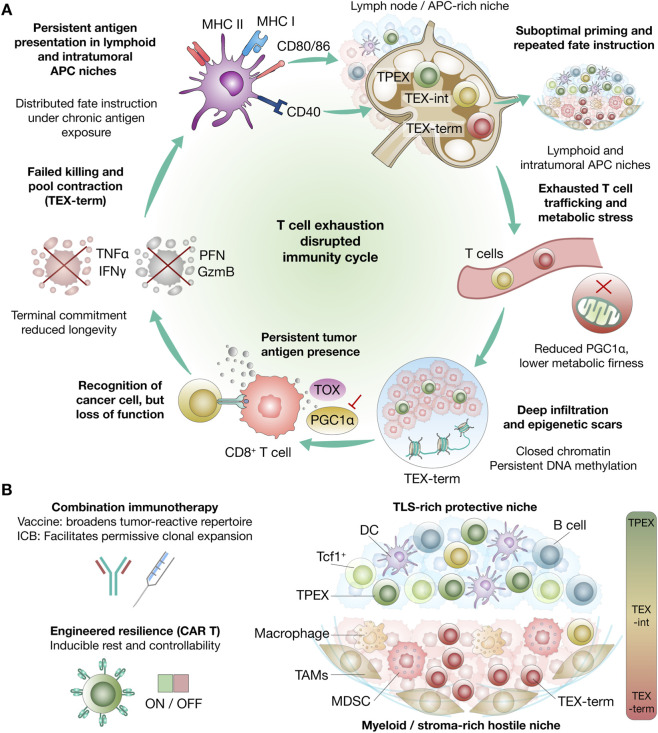
T cell exhaustion as a dynamic and therapeutically modulable state system in cancer immunity. **(A)** Schematic model of the disrupted immunity cycle associated with T cell exhaustion under persistent tumor antigen exposure. Chronic antigen presentation and fate instruction across lymphoid and intratumoral antigen-presenting cell niches promote induction of exhaustion-associated programs, including TOX-linked differentiation, during suboptimal priming and repeated antigen exposure. This process supports the emergence of TPEX, TEX-int, and TEX-term. As exhausted T cells traffic into tumors, persistent antigen exposure, metabolic stress, reduced mitochondrial fitness, and suppression of mitochondrial biogenesis programs, including reduced PGC1α activity, further constrain function and persistence. Deep tumor infiltration is associated with epigenetic scarring, including closed chromatin states and persistent DNA methylation, which can limit reinvigoration. Although exhausted CD8^+^ T cells may still recognize tumor cells, they progressively lose effector function, including cytokine production and cytotoxic mediator expression, ultimately leading to failed killing, pool contraction, terminal commitment, and reduced longevity. **(B)** Therapeutic and spatial framework of exhaustion control. Combination immunotherapy, including vaccination and ICB, may broaden the tumor-reactive repertoire and facilitate permissive clonal expansion, whereas engineered cell therapies such as CAR T cells can incorporate inducible rest and ON/OFF control circuits to enhance resilience and controllability. Spatially, progenitor-like exhausted T cells are preferentially represented in protective niches enriched in TLS, DCs, B cells, and Tcf1^+^ cells, whereas more terminally exhausted states accumulate in hostile myeloid- and stroma-rich niches containing TAMs, MDSCs, and suppressive stromal elements. Together, these features position exhaustion as a structured, spatially organized, and partially controllable state landscape with direct relevance to cancer immunotherapy. CAR, chimeric antigen receptor; DC, dendritic cell; GzmB, granzyme B; ICB, immune checkpoint blockade; IFNγ, interferon gamma; MDSC, myeloid-derived suppressor cell; MHC, major histocompatibility complex; PFN, perforin; PGC1α, peroxisome proliferator-activated receptor gamma coactivator 1-alpha; TAM, tumor-associated macrophage; TEX-int, intermediate exhausted T cell; TEX-term, terminally exhausted T cell; TPEX, progenitor exhausted T cell; TLS, tertiary lymphoid structure; TNFα, tumor necrosis factor alpha; TOX, thymocyte selection-associated high mobility group box protein.

Within this landscape, many datasets support a recurrent organization extending from progenitor exhausted T cells (TPEX), through intermediate exhausted states (TEX-int), to terminally exhausted states (TEX-term) (Beltra et al., 2020; Miller et al., 2019; Rahim et al., 2023) (Figure 1A). However, this TPEX–TEX-int–TEX-term framework should be interpreted as a conceptual scaffold rather than a strictly universal map across species and tumor settings. It was defined most clearly in murine chronic stimulation models, and although human tumors contain broadly analogous progenitor-like and more differentiated exhausted populations, their correspondence to murine subsets is not always exact (Utzschneider et al., 2016; Beltra et al., 2020; Siddiqui et al., 2019). TPEX cells are typically enriched for progenitor-associated features such as TCF1/TCF7 expression and are relatively depleted of markers associated with terminal commitment, including TIM-3/HAVCR2, while remaining part of an exhausted lineage defined by chronic antigen exposure and inhibitory receptor expression (Im et al., 2016; Miller et al., 2019). In tumors, markers such as SLAMF6 may enrich for this progenitor-like compartment in some settings; however, this association should be interpreted cautiously, as recent studies indicate that SLAMF6 can also function as an inhibitory receptor that restrains T cell activation and limits antitumor immunity, rather than serving as a uniformly supportive marker of progenitor exhausted T cell biology (Li et al., 2026; Hajaj et al., 2020; Siddiqui et al., 2019). In human cancers, additional variability arises from interpatient heterogeneity, prior treatment exposure, anatomical sampling site, and overlap with activation or tissue-resident memory programs, all of which can blur state boundaries and complicate one-to-one alignment with murine Tex subsets (Oliveira et al., 2021; Yost et al., 2019; Virassamy et al., 2023). Functionally, TPEX cells are most consistently linked to proliferative competence and the capacity to generate downstream exhausted progeny under persistent antigenic stimulation, whereas TEX-term cells show broader inhibitory receptor co-expression and transcriptional programs associated with deeper differentiation and more limited expansion potential. TEX-int occupies the continuum between these poles and may include dividing, effector-leaning, or progressively constrained states depending on context and sampling depth (Im et al., 2016; Beltra et al., 2020). Cancer-related experimental support comes from tumor models showing that the exhausted compartment is not functionally uniform: Miller et al. demonstrated that progenitor exhausted CD8^+^ TILs differ functionally from more terminal subsets and preferentially respond to PD-1 blockade (Miller et al., 2019), whereas Siddiqui et al. showed that intratumoral Tcf1^+^PD-1^+^ stem-like T cells generate differentiated exhausted progeny and support immunotherapy-driven expansion (Siddiqui et al., 2019). These findings anchor the state-space model in tumor immunotherapy by showing that exhausted-state organization has direct functional and therapeutic consequences. The defining molecular, functional, epigenetic, metabolic, spatial, and therapeutic features of the major exhausted CD8^+^ T cell states are summarized in Table 1.

**TABLE 1:** Defining features of major exhausted CD8^+^ T cell states in cancer.

Feature	TPEX	TEX-int	TEX-term
Representative markers/transcriptional features	TCF1/TCF7^high^, PD-1^+^, relative depletion of terminal markers such as TIM-3/HAVCR2; SLAMF6 may enrich this compartment in some settings but should not be interpreted as a uniformly supportive functional marker	Intermediate differentiation program; partial acquisition of terminal markers; context-dependent proliferative and effector-associated signatures	High inhibitory receptor co-expression, including TIM-3/HAVCR2; TOX-associated terminal program
Functional properties	Proliferative competence, self-renewal potential, source of downstream exhausted progeny	Transitional state linking progenitor and terminal compartments; variable effector output and proliferative behavior	Limited proliferative capacity, reduced polyfunctionality, impaired cytotoxic persistence
Epigenetic and metabolic constraints	Less terminally fixed chromatin state; relatively preserved mitochondrial fitness compared with terminal states	Increasing regulatory constraint; progressive metabolic strain	More fixed chromatin landscape, persistent DNA methylation, reduced PGC1α activity, lower metabolic fitness
Spatial associations	Often enriched in TLS-like, APC-rich, or lymphoid-like niches	Distributed across tumor regions; may reflect active transition under persistent antigen exposure	Often enriched in myeloid-rich, stromal, metabolically stressed, and suppressive niches
Therapeutic relevance	Major reservoir for response to PD-1 blockade; key determinant of durable benefit	May contribute to on-treatment expansion and short-term function	Less likely to sustain durable reinvigoration; often associated with partial or transient functional rescue
Predominant evidence base	Murine framework + supportive human data	Predominantly murine; limited and context-dependent human inference	Murine framework + supportive human data
References	Beltra et al. (2020), Miller et al. (2019), Siddiqui et al. (2019), Oliveira et al. (2021)	Miller et al. (2019), Beltra et al. (2020)	Oliveira et al. (2021), Miller et al. (2019), Beltra et al. (2020)

The TPEX–TEX-int–TEX-term framework is defined most rigorously in murine models of chronic antigen exposure and tumor immunity. Human tumors contain broadly analogous progenitor-like and more differentiated exhausted CD8^+^ T cell populations, but marker combinations, state boundaries, and developmental relationships are less uniform and are often inferred from cross-sectional single-cell, clonotypic, and phenotypic analyses rather than direct lineage resolution.

TPEX, progenitor exhausted T cells; TEX-int, intermediate exhausted T cells; TEX-term, terminally exhausted T cells; TCF1/TCF7, T cell factor 1/transcription factor 7; PD-1, programmed cell death protein 1; TIM-3, T cell immunoglobulin and mucin-domain containing 3; HAVCR2, hepatitis A virus cellular receptor 2; SLAMF6, signaling lymphocytic activation molecule family member 6; TOX, thymocyte selection-associated high mobility group box; PGC1α, peroxisome proliferator-activated receptor gamma coactivator 1-alpha; TLS, tertiary lymphoid structures; APC, antigen-presenting cells.

Single-cell approaches have made this framework increasingly quantifiable. ScRNA-seq can resolve exhausted subpopulations without reliance on preselected marker gates, often identifying minority progenitor-like clusters alongside larger terminal compartments (Satpathy et al., 2019). Integration with TCR sequencing links these states to clonal structure and allows related clonotypes to be tracked across phenotypic regions, including during immune checkpoint blockade in human tumors (Yost et al., 2019; Oliveira et al., 2021). Chromatin accessibility profiling adds a regulatory dimension that often distinguishes progenitor-like and terminal compartments beyond what would be expected from transient activation alone (Sen et al., 2016). Recent single-cell studies in human tumors, including pan-cancer analyses, further strengthen this framework by identifying multiple transcriptionally distinct exhausted CD8^+^ T cell states that recur across malignancies while differing in relative abundance, differentiation bias, and tumor context, reinforcing the view that human T cell exhaustion is organized as a heterogeneous state system rather than a single dysfunctional endpoint (Zheng et al., 2021; Zhang et al., 2022; Tooley et al., 2024). Together, these approaches support the view that exhaustion is organized as a structured continuum, although claims about developmental directionality are strongest when transcriptional, clonal, and epigenomic evidence converge rather than when they rely on trajectory inference alone (La Manno et al., 2018; Beltra et al., 2020; Saelens et al., 2019). This caution is particularly important in human tumors, where most datasets remain cross-sectional and where inferred trajectories may reflect state similarity, spatial compartmentalization, treatment-associated remodeling, or clonal replacement rather than directly observed lineage progression (Yost et al., 2019).

This framework also helps explain why reversal of exhaustion is often limited. Rather than invoking absolute irreversibility, it is more useful to consider that some regions of exhausted state space may become progressively constrained, such that clinically relevant perturbations can transiently improve function without durably restoring progenitor-like or memory-like identity (Abdel-Hakeem et al., 2021; Pauken et al., 2016; Schietinger et al., 2016). In this view, movement toward more terminal exhausted states is commonly observed under sustained antigenic stress, whereas sustained reverse remodeling appears more restricted and likely depends on context, timing, and the availability of upstream progenitor-like reservoirs (Philip et al., 2017; Beltra et al., 2020).

Across studies, several conclusions are relatively consistent: exhausted PD-1^+^ compartments contain structured heterogeneity; less differentiated TCF1-enriched subsets recurrently coexist with more differentiated populations; and state composition can be linked, at least in part, to clonal structure and treatment-associated expansion (Zheng et al., 2021; Im et al., 2016; Yost et al., 2019). At the same time, marker definitions, state boundaries, and inferred topologies vary across tumor types, tissue compartments, and analytical platforms. In humans, this variability is often amplified by tumor-specific ecology and by partial overlap between exhaustion, activation, and residency programs, making rigid state assignments less robust than simplified nomenclature may imply (Barsch et al., 2022; Li et al., 2019). For this reason, the most robust interpretations rely on explicit state definitions and cross-modal concordance rather than on over-interpretation of any single embedding or trajectory model (Andreatta et al., 2021; Saelens et al., 2019). Taken together, this state-based framework provides the conceptual basis for understanding how exhaustion is not only organized, but also actively stabilized under chronic antigen exposure.

## TOX and survival-driven fate control

Within this broader state architecture, the next question is what regulatory programs actively stabilize exhausted fate rather than merely accompany it. TOX is most informative when interpreted as part of a survival-oriented fate program induced by persistent antigenic stimulation rather than as a simple marker of dysfunction. Across chronic infection models and multiple tumor settings, repeated TCR engagement promotes exhaustion-associated transcriptional programs in which TOX is consistently upregulated in exhausted CD8^+^ T cells and contributes to consolidation of the exhausted state (Alfei et al., 2019; Yao et al., 2019) (Figure 1A). A plausible mechanistic framework is that chronic stimulation sustains calcium–calcineurin signaling and NFAT activity under conditions of limited productive AP-1 coupling, thereby favoring a hyporesponsive but persistence-compatible transcriptional program that includes TOX and related regulators such as NR4A family members (Martinez et al., 2015; Chen et al., 2019). In this context, TOX is best viewed not as an isolated driver but as part of a broader regulatory circuit that converts sustained stimulation into a durable state compatible with continued survival under ongoing antigen exposure (Scott et al., 2019; Alfei et al., 2019).

This framework helps explain findings from perturbation studies. Reducing TOX activity in chronic settings can decrease inhibitory receptor expression and transiently enhance cytokine production or polyfunctionality, yet these early gains are often accompanied by reduced long-term persistence, contraction of responding populations, or increased immunopathology (Alfei et al., 2019; Yao et al., 2019; Khan et al., 2019). These patterns suggest that TOX-linked programs do not simply suppress effector function; rather, they impose constraints that limit overstimulation-associated damage while preserving population continuity under persistent antigenic stress (Wherry and Kurachi, 2015). Cancer-related experimental data support this interpretation. In hepatocellular carcinoma, TOX was shown to promote exhaustion of antitumor CD8^+^ T cells by sustaining PD-1 through endocytic recycling, and TOX downregulation enhanced effector function. In bladder cancer, TOX-expressing PD-1^high^ CD8^+^ TILs defined a terminally exhausted, tumor-reactive population that could be more effectively reinvigorated by combined PD-1 and TIGIT blockade (Wang et al., 2019; Han et al., 2021). These findings indicate that TOX is not merely a passive exhaustion marker in tumors, but a functional regulator of terminal dysfunction and checkpoint responsiveness, directly linking TOX biology to the design of combination immunotherapy strategies.

A key implication is that the relationship between function and persistence is not all-or-none. Different components of the exhausted phenotype, including inhibitory receptor expression, proliferative competence, cytokine production, and lineage stability, may be differentially regulated and need not change in parallel. This raises the possibility that partial modulation of TOX-linked circuitry could improve selected functional outputs without completely compromising persistence, although current evidence indicates that such effects are highly context-dependent and influenced by antigen load, inflammatory tone, tissue environment, and timing of perturbation (Lynn et al., 2019; Khan et al., 2019).

For this reason, TOX-linked fate control is best evaluated in time-resolved rather than endpoint terms. Interventions that blunt TOX-associated programs may improve early effector readouts while impairing late maintenance, manifested as diminished clonal persistence, reduced proliferative reserve, or failure to sustain responses under continued antigen exposure. In this regard, preservation *versus* rapid consumption of progenitor-like exhausted pools may provide an especially informative readout of the survival–function trade-off, helping distinguish transient functional enhancement from durable therapeutic benefit (Alfei et al., 2019; Siddiqui et al., 2019).

Overall, TOX should be considered one component of a broader regulatory network that stabilizes exhausted fate under chronic stimulation. The translational challenge is therefore not whether TOX is intrinsically beneficial or detrimental, but whether TOX-linked programs can be modulated in ways that expand functional capacity without undermining the fitness and persistence required for sustained antitumor responses (Chen et al., 2019; Man et al., 2017). If TOX-linked programs help explain why exhausted cells persist, the next question is why therapeutic rescue so often remains incomplete even when inhibitory signaling is relieved.

## Epigenetic limits of reinvigoration

One major answer lies in the epigenetic architecture that constrains how far reinvigoration can proceed. Checkpoint blockade can restore selected effector functions in exhausted CD8^+^ T cells, but multiple studies indicate that this rebound often occurs without full reversion of cellular identity (Pauken et al., 2016; Sen et al., 2016). A conservative interpretation is that chronic antigen stimulation establishes relatively stable epigenetic features, including changes in chromatin accessibility, enhancer usage, and DNA methylation, that restrict the transcriptional programs available after inhibitory signaling is relieved (Figure 1A). Thus, blockade can increase functional activity without fully reopening the identity trajectories associated with naïve, effector, or durable memory states (Mognol et al., 2017; Philip et al., 2017; Bautista and Lopez-Cortes, 2025).

Consistent with this view, exhausted T cells in chronic infection and cancer reproducibly display chromatin-accessibility landscapes that differ from those of canonical effector and memory cells, with regulatory elements linked to exhaustion-associated programs and sustained inhibitory receptor expression (Sen et al., 2016; Satpathy et al., 2019). Transition into exhaustion is also accompanied by DNA methylation remodeling, including *de novo* methylation at loci associated with effector function, trafficking, and progenitor-related features. In several systems, these methylation patterns persist despite functional improvement after PD-1/PD-L1 blockade, supporting the idea that checkpoint therapy can alter output without broadly dismantling the regulatory architecture that stabilizes exhausted identity (Youngblood et al., 2019; Abdel-Hakeem et al., 2021). At the same time, not all epigenetic features are fixed, and the balance between stable and dynamic elements likely varies across exhausted states and tumor contexts (Ocaña-Paredes et al., 2024). Cancer-related experimental support for this concept comes from studies showing that epigenetic fixation constrains therapeutic rescue rather than simply accompanying it. In chronically stimulated tumor-reactive CD8^+^ T cells, PD-1 blockade can restore selected functions without erasing the exhaustion-specific chromatin landscape, and disruption of *de novo* DNA methylation programs can enhance responsiveness to checkpoint therapy by limiting acquisition of the more fixed exhausted state (Miller et al., 2019; Oliveira et al., 2021). Together, these findings indicate that reinvigoration and true identity resetting are not equivalent processes, and that durable therapeutic benefit may require direct modification of the epigenetic architecture that stabilizes dysfunction.

These observations help explain why reinvigoration is often incomplete or transient. In experimental models, checkpoint blockade commonly increases proliferation and effector-associated transcripts, but these changes may be dominated by activation and cell-cycle programs rather than by durable convergence toward memory-like regulatory states (Huang et al., 2017; Mognol et al., 2017). When antigen persists, reinvigorated cells can reacquire exhaustion-associated features, and even after antigen withdrawal, stable protective memory remains limited in some settings. In patients, anti–PD-1 responses have in some cohorts been associated with expansion of newly detected clonotypes rather than broad recovery of pre-existing exhausted clones, consistent with restricted reprogrammability of chronically imprinted populations (Yost et al., 2019; Pauken et al., 2016). However, other studies support expansion of pre-existing tumor-infiltrating clonotypes and substantial functional improvement within exhausted compartments, indicating that reinvigoration can be meaningful even without complete identity resetting (Oliveira et al., 2021; Caushi et al., 2021).

A related implication is that exhausted cells may retain persistent regulatory features after chronic stimulation, often described as chromatin scars, that continue to mark exhaustion-linked loci even after antigen withdrawal (Abdel-Hakeem et al., 2021; Sen et al., 2016). This supports a stringent definition of true reprogramming: not merely transient functional rescue, but coordinated and durable remodeling of identity-defining regulatory elements. Evidence for deeper resetting would include contraction of exhaustion-specific enhancer accessibility, reversal of exhaustion-linked DNA methylation at key fate and effector loci, and restoration of long-term recall capacity and persistence under antigen-free conditions (Youngblood et al., 2019; Sen et al., 2016).

Preclinical studies further suggest that limiting acquisition of exhaustion-associated methylation, or partially relaxing epigenetic constraints, can enhance responses to PD-1 pathway blockade, supporting the idea that epigenetic architecture is functionally important rather than purely correlative (Ghoneim et al., 2017; Belk et al., 2022). Even so, resetting is unlikely to be uniform across exhausted states or disease settings, and improved effector function can coexist with persistent identity constraints (Zheng et al., 2021; Pauken et al., 2016). Overall, checkpoint blockade is most conservatively interpreted as shifting function within an epigenetically constrained landscape, whereas durable identity reprogramming likely requires additional conditions or direct remodeling of the regulatory programs that stabilize exhaustion (Satpathy et al., 2019; Belk et al., 2022). These limits on reprogramming help explain why durable benefit often depends not on wholesale reversal of exhaustion, but on whether less differentiated compartments remain available to renew the response.

## Progenitor exhausted T cells sustain response

This places progenitor exhausted T cells at the center of therapeutic durability. Durable benefit from checkpoint blockade is often better explained by changes in the composition and dynamics of exhausted CD8^+^ T cell states than by uniform reversal of terminal exhaustion across the entire compartment. Across chronic infection models and multiple tumor settings, studies support the existence of a less differentiated progenitor exhausted population (TPEX) that retains proliferative potential, persists under chronic antigen exposure, and gives rise to intermediate and terminally exhausted progeny (Im et al., 2016; Utzschneider et al., 2016; Wu et al., 2016). This framework suggests that sustained therapeutic benefit depends largely on the presence of an upstream renewal-competent compartment capable of repeated expansion under inhibitory relief, rather than on terminal populations with limited proliferative reserve (Sade-Feldman et al., 2018; Im et al., 2016; Zheng et al., 2021).

This compartment is commonly identified by sustained TCF1 (TCF7) expression within the PD-1^+^ antigen-experienced pool, often together with phenotypic features of partial differentiation, including inhibitory receptor expression that is present but not maximal and relative depletion of markers associated with terminal commitment (Im et al., 2016). Although often described as stem-like, this designation is best interpreted operationally. In many systems, these cells display greater proliferative competence upon stimulation and generate downstream states with stronger immediate effector activity but reduced long-term expansion capacity. After checkpoint blockade, expanding populations in blood and tumor are frequently enriched for cells mapping to this progenitor-like region and their immediate descendants, consistent with a response architecture in which upstream compartments drive proliferative expansion, whereas downstream compartments contribute a larger share of short-lived effector output (Sade-Feldman et al., 2018). However, treatment-associated changes can also reflect recruitment of newly detected clonotypes, redistribution between compartments, or differential survival, and these possibilities are best resolved through clonal tracking and longitudinal sampling (Yost et al., 2019). Cancer-related experimental evidence strongly supports the view that TPEX cells function as a renewal-competent reservoir for sustained antitumor immunity. In tumor systems, Siddiqui et al. showed that intratumoral Tcf1^+^PD-1^+^ stem-like CD8^+^ T cells mediate proliferative responses to immunotherapy and seed downstream exhausted progeny (Siddiqui et al., 2019), whereas Miller et al. demonstrated that this progenitor exhausted subset, rather than terminally exhausted TILs, is most capable of sustaining tumor control under PD-1 blockade (Miller et al., 2019). These findings indicate that durable therapeutic benefit depends not simply on reinvigorating exhausted cells in bulk, but on preserving and engaging the upstream compartment that continuously renews the antitumor response.

The broader organization of exhaustion further supports this model as a continuum in which progenitor-like states occupy an upstream region, intermediate states bridge toward more terminal configurations, and progression is associated with sustained antigenic stimulation (Beltra et al., 2020; Schietinger et al., 2016). Within this framework, maintenance of a progenitor-like compartment is a plausible determinant of response durability. In contrast, depletion of this compartment is associated with downstream-skewed state distributions and diminished capacity for repeated proliferative surges even when inhibitory signaling is relieved (Utzschneider et al., 2016; Beltra et al., 2020). Thus, transient functional improvement may still occur without durable benefit if the renewal-competent pool is numerically scarce, spatially restricted, or functionally compromised.

From a translational perspective, the status of this compartment is best viewed as a composite and context-dependent feature rather than a standalone biomarker. Informative readouts may include the frequency of TCF1^+^ cells within the PD-1^+^ compartment, the balance between progenitor-like and terminally differentiated exhausted states, and evidence that early on-treatment expansion is clonally connected to the progenitor-like pool (Sade-Feldman et al., 2018; Wu et al., 2016; Thommen et al., 2018). Although these measures are unlikely to support deterministic prediction, they provide a biologically grounded framework for assessing whether a renewal-competent population is present and measurably engaged during therapy. Overall, progenitor exhausted T cells are important not only as a distinct phenotypic subset but as a renewable source for therapy-associated expansion, making the size, accessibility, and functional competence of the TPEX compartment central determinants of sustained antitumor benefit. However, even a renewal-competent compartment remains subject to the metabolic liabilities imposed by the tumor microenvironment.

## Bioenergetic constraints on T cell persistence

Accordingly, therapeutic responsiveness depends not only on state identity, but also on whether cellular metabolism can sustain persistence and expansion. Metabolic stress in the tumor microenvironment (TME) can strongly influence whether an antigen-experienced CD8^+^ T cell maintains a renewal-competent program or shifts toward more constrained exhausted states (Scharping et al., 2016) (Figure 1A). Across tumor datasets and chronic-stimulation systems, more dysfunctional or terminally exhausted populations are frequently associated with reduced mitochondrial fitness, including lower respiratory capacity, diminished spare respiratory reserve, and transcriptional signatures of metabolic strain (Bengsch et al., 2016). Because chronically stimulated cells can retain evidence of ongoing signaling while exhibiting limited proliferative competence, these patterns are not readily explained by insufficient activation alone. A conservative interpretation is that persistent stimulation in the TME imposes energetic and biosynthetic demands that become increasingly difficult to meet, thereby narrowing the conditions under which sustained expansion and renewal-associated programs can be maintained (Chang et al., 2015; Vardhana et al., 2020).

One recurring feature of exhausted-like states is impaired oxidative metabolism. In several experimental settings, chronic stimulation is associated with reduced oxygen consumption, altered coupling between respiration and ATP production, and broader signs of energetic stress (Bengsch et al., 2016; Vardhana et al., 2020). Because sustained proliferation depends on ATP availability, redox balance, and biosynthetic support, these limitations provide a coherent explanation for why continued cell-cycle entry becomes difficult even in the presence of antigen and inflammatory cues. Consistent with this idea, acute restriction of mitochondrial respiration reduces proliferative output and shifts gene-expression programs away from renewal-associated states, whereas interventions that limit mitochondrial oxidative stress or improve mitochondrial capacity can preserve proliferative competence and modestly improve selected effector functions (Fisicaro et al., 2017; Scharping et al., 2016). However, improved bioenergetics should not be equated with full reversal of exhausted identity. Cancer-related experimental evidence strongly supports this constraint. Bengsch et al. showed that exhausted CD8^+^ T cells display metabolic insufficiency linked to mitochondrial dysfunction (Bengsch et al., 2016), whereas Scharping et al. demonstrated in tumor-reactive T cells that chronic stimulation promotes mitochondrial depolarization, oxidative stress, and loss of metabolic fitness, and that restoring mitochondrial biogenesis can partially improve T cell function (Scharping et al., 2016). Together, these findings indicate that metabolic failure is not simply a secondary correlate of exhaustion, but a mechanistic limiter of proliferative persistence and antitumor efficacy, making mitochondrial fitness a therapeutically relevant target in cancer immunotherapy.

Constrained mitochondrial biogenesis may further contribute to this limitation. Compared with lymphoid counterparts, tumor-infiltrating CD8^+^ T cells often show reduced expression of regulators of oxidative metabolism and mitochondrial renewal, including PGC1α, together with lower mitochondrial mass and weaker proliferative features (Dumauthioz et al., 2021). Signaling environments that maintain Akt activity and suppress Foxo-family transcriptional programs provide one plausible route by which chronic stimulation and tumor-derived cues restrain mitochondrial renewal (Kim et al., 2013; Fraietta et al., 2018). In line with this, pharmacologic or genetic strategies that enhance mitochondrial biogenesis can increase respiratory reserve and support T cell function in defined settings, although these improvements remain compatible with persistent exhaustion-associated regulatory constraints (Dumauthioz et al., 2021; Scharping et al., 2016).

Nutrient limitation adds another layer of restriction. Tumor and myeloid cells can reduce local availability of key metabolites, including amino acids such as tryptophan, thereby engaging stress-response pathways such as GCN2, dampening mTOR-dependent anabolic signaling, and slowing cell-cycle progression (Uyttenhove et al., 2003; Munn et al., 2005). Tryptophan catabolism also generates kynurenine, which can signal through the aryl hydrocarbon receptor and further restrain effector programs (Opitz et al., 2011; Bessede et al., 2014). More broadly, hypoxia, adenosine accumulation, glucose competition, and oxidative stress converge in solid tumors to limit respiration, glycolytic support, and redox homeostasis, reinforcing dysfunctional or stress-adapted states (Ohta and Sitkovsky, 2001; Hatfield et al., 2015; López-Cortés et al., 2022). These pressures are best viewed as interacting boundary conditions rather than as isolated deterministic switches.

Taken together, mitochondrial fitness, nutrient availability, and redox balance are repeatedly associated with exhausted-state composition and with limits on proliferative persistence. Their effects likely operate alongside antigen load and inhibitory signaling to narrow the range of accessible T cell states, particularly those capable of sustained expansion during immunotherapy (Kim and DeBerardinis, 2019; Zheng et al., 2021; Scharping et al., 2016). Yet exhausted states are not distributed randomly in tissue, and metabolic liabilities themselves unfold within specific spatial niches.

## Spatial organization of exhausted T cells

A spatial perspective, therefore, complements state-based and metabolic models by asking where different exhausted populations are most likely to persist, expand, or fail. Spatial organization adds an important layer to the interpretation of exhausted T cell states. Within a single tumor, T cells are distributed across microenvironments that differ in antigen-presenting cell density, stromal architecture, vascular access, oxygenation, nutrient availability, and the balance between supportive and suppressive immune populations. As a result, exhausted-state composition is often not spatially uniform, and phenotypes that appear similar in bulk can resolve into distinct neighborhood-specific distributions when tissue context is preserved (Schürch et al., 2020; Sudmeier et al., 2022). Thus, where an exhausted state is observed can be as informative as which state is observed, particularly when comparing samples from different tumor regions or time points.

Tertiary lymphoid structures (TLS) represent one recurrent microenvironment relevant to exhaustion biology. These ectopic lymphoid aggregates can contain B cells, T cells, antigen-presenting cells, and specialized vascular or stromal features, providing a context in which antigen presentation and lymphocyte cross-talk are locally organized (Sautès-Fridman et al., 2019; Astorri et al., 2010). In several settings, TLS-rich regions have been associated with broader distributions of T cell phenotypes, including progenitor-like or less terminally differentiated subsets, relative to adjacent tumor regions enriched for more terminal programs (Jansen et al., 2019; Li et al., 2025). However, TLS should not be treated as a universal proxy for effective antitumor immunity, because its significance depends on factors such as maturation state, cellular composition, and proximity to tumor nests (Siliņa et al., 2018) (Figure 1B).

By contrast, more terminal exhaustion-associated phenotypes are often found in myeloid-rich and stroma-reactive neighborhoods (Bautista and López-Cortés, 2025b). These regions commonly contain tumor-associated macrophages, other myeloid populations, fibroblast programs, and extracellular matrix features that coincide with altered T cell access, retention, and signaling context (Pelka et al., 2021; Enfield et al., 2024). They are frequently associated with inhibitory ligands, inflammatory mediators, and tissue stress signatures, and often co-localize with T cell states displaying deeper exhaustion-associated features (Schürch et al., 2020; Pelka et al., 2021). Even so, these associations should be interpreted descriptively rather than deterministically, because spatial co-occurrence alone does not establish a mechanism or prove that a given niche imposes a fixed fate. Cancer-related experimental evidence strongly supports the view that exhausted-state composition is spatially structured within tumors. In human colorectal cancer, Schürch et al. identified multicellular neighborhoods in which distinct immune and stromal architectures were associated with differential immune states (Schürch et al., 2020), whereas Pelka et al. showed that tumor-reactive and dysfunctional T cell programs are spatially linked to specific epithelial, myeloid, and stromal contexts (Pelka et al., 2021). Together, these findings indicate that exhaustion is not simply a cell-intrinsic differentiation problem, but a tissue-organized phenomenon in which local niche structure helps determine which T cell states are sustained, excluded, or terminally reinforced. This matters for cancer immunotherapy because therapeutic efficacy may depend not only on the presence of tumor-reactive T cells, but also on whether they occupy spatially permissive *versus* suppressive neighborhoods. Recent evidence further suggests that neural injury and neuroimmune signaling may constitute an additional layer of niche organization within tumors, as degenerating neurons can remodel local immune architecture, promote immunosuppressive inflammatory programs, and contribute to tumor-permissive microenvironments, reinforcing the view that exhaustion is shaped not only by immune and stromal context but also by innervated niche structure (Bautista et al., 2025a; Baruch et al., 2025).

A key point is that spatial organization does not by itself define directionality or causality. Related clonotypes can be detected across distinct neighborhoods, suggesting that spatial context may partition shared lineages into different state distributions rather than generate entirely separate repertoires (Zheng et al., 2021). Likewise, regional differences may reflect recruitment, retention, local proliferation, or differential survival as much as local instruction. For this reason, spatial ecology is most informative when treated as a quantitative problem: which states are enriched in which neighborhoods, how reproducible those associations are across tumor contexts, and whether longitudinal changes in niche composition track with changes in state distribution, proliferation, and clonal occupancy (Schürch et al., 2020; Yost et al., 2019).

Recent spatial transcriptomic, multiplex imaging, and integrated single-cell approaches have made these questions increasingly tractable by defining microdomains based on co-localized cell types, coordinated gene programs, and inferred interaction networks (Li et al., 2025; Jansen et al., 2019). Together, these studies support a cautious but consistent conclusion: neighborhood structure frequently covaries with exhausted-state composition and may constrain which T cell states are locally sustained, even if it does not uniquely determine fate (Longo et al., 2021). Overall, a spatial ecology framework helps position exhaustion as a tissue-distributed phenomenon and provides a more rigorous basis for comparing exhausted T cell states across tumors, regions, and treatment contexts. Taken together, these state, regulatory, metabolic, and spatial constraints define the biological boundaries within which therapeutic modulation of exhaustion must operate.

## Therapeutic control of exhaustion dynamics

**FIGURE 2:**
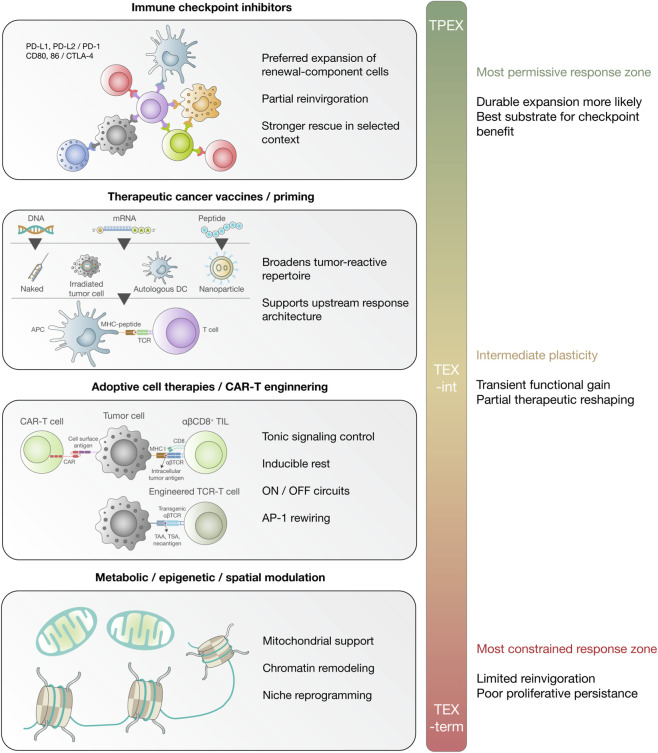
State-specific therapeutic intervention points across the exhausted CD8^+^ T cell continuum in cancer. The central vertical axis depicts the exhausted CD8^+^ T cell continuum extending from progenitor exhausted T cells (TPEX), through intermediate exhausted T cells (TEX-int), to terminally exhausted T cells (TEX-term), emphasizing that exhausted states are not therapeutically equivalent. The left side summarizes major therapeutic strategies positioned according to the region of the continuum they are most likely to influence. Immune checkpoint inhibitors are shown as acting preferentially on renewal-competent and partially constrained compartments, where they are associated with preferred expansion of upstream exhausted cells, partial reinvigoration, and, in selected settings, stronger rescue with combined blockade. Therapeutic cancer vaccines and priming strategies are shown as upstream interventions that broaden the tumor-reactive repertoire and support response architecture. Adoptive cell therapies, including CAR-T engineering, are depicted as parallel strategies designed to reduce tonic signaling, permit inducible rest, and incorporate ON/OFF control circuits or transcriptional rewiring to limit exhaustion-like remodeling. Metabolic, epigenetic, and spatial modulation are shown as complementary approaches intended to improve mitochondrial fitness, relax stabilizing chromatin constraints, or reprogram suppressive niches. The right side summarizes the expected therapeutic outputs and major limitations across the continuum, highlighting that durable expansion is most likely in permissive upper states, that intermediate states may support transient functional gain and partial reshaping, and that more terminally exhausted states remain limited by reduced proliferative persistence, epigenetic fixation, metabolic insufficiency, and hostile tissue context. Together, the figure illustrates that immunotherapies act on distinct regions of the exhausted-state landscape and that therapeutic efficacy depends on both state composition and the biological constraints that limit reversibility. CAR-T, chimeric antigen receptor T cells; CTLA-4, cytotoxic T-lymphocyte-associated protein 4; PD-1, programmed cell death protein 1; PD-L1, programmed death-ligand 1; TPEX, progenitor exhausted T cells; TEX-int, intermediate exhausted T cells; TEX-term, terminally exhausted T cells; TIGIT, T cell immunoreceptor with Ig and ITIM domains.

From this perspective, therapeutic intervention is best understood not as simple rescue, but as an effort to reshape exhausted-state dynamics within definable biological limits (Figure 2). Clinical translation of exhaustion biology is increasingly framed as a problem of coordinating antigenic stimulation, relief of inhibitory signaling, and toxicity management. A practical limitation of PD-1/PD-L1 blockade is that measurable functional improvement does not always translate into durable tumor control, in part because the pool of tumor-reactive T cells may be numerically or functionally insufficient to sustain repeated waves of productive expansion (Huang et al., 2017; Pauken et al., 2016). Within this framework, therapeutic vaccination is commonly positioned as an upstream intervention that broadens or amplifies tumor-reactive repertoires, whereas checkpoint blockade provides a permissive context for clonal expansion and effector deployment rather than generating new antigen-specificities on its own (Rojas et al., 2023; Khleif and Gupta, 2025) (Figures 1B, 2). A conservative measure of success is therefore proximal and measurable: increased frequency, breadth, or functional quality of tumor-reactive T cells, together with evidence of expansion or trafficking under checkpoint relief without disproportionate immune toxicity (Ott et al., 2017; Larkin et al., 2015).

A central translational uncertainty concerns sequencing: whether vaccination should precede PD-1 blockade, be initiated concurrently, or be layered after checkpoint therapy has begun. Each sequence implies distinct, testable hypotheses about how inhibitory relief interacts with priming and with the maturation state of the responding pool (D’Alise et al., 2019; Guo et al., 2022). A priming-first approach is conceptually consistent with applying inhibitory relief to an antigen-experienced population shaped under vaccine-driven instruction, whereas initiating PD-1 blockade early or without adequate priming could preferentially expand bystander or suboptimal populations or generate expansion that is not sustained once the inflammatory context contracts. Conversely, delaying PD-1 blockade may allow continued stimulation in an inhibitory environment to shift tumor-reactive clones toward more constrained configurations, narrowing the window in which expansion is coupled to tumor control. Similar considerations apply to prime/boost strategies and to multimodality settings involving surgery, radiation, cytotoxic therapy, or steroid exposure, all of which can alter antigen availability, inflammatory tone, and lymphocyte composition (Galluzzi et al., 2015; Twyman-Saint Victor et al., 2015; Hailemichael et al., 2013).

Accordingly, timing is best treated not simply as an empirical variable anchored to measurable kinetics, but as a systems-level biological dimension governed by circadian organization and zeitgeber-dependent entrainment, with growing implications for chronotherapy and circadian precision medicine (Pérez-Villa et al., 2023; Bautista and López-Cortés, 2025a; Bautista et al., 2025b). Importantly, this statement should not be interpreted as evidence that circadian timing directly regulates T cell exhaustion itself. Exhaustion is a prolonged differentiation process shaped by persistent antigen stimulation, inhibitory signaling, epigenetic remodeling, metabolic stress, and tissue context, and direct evidence that circadian organization governs discrete TPEX, TEX-int, or TEX-term states remains limited. Rather, circadian timing is best positioned here as a contextual modifier of exhaustion-relevant immune processes, including antigen presentation, T cell priming, vaccination responses, intratumoral CD8^+^ T cell abundance or function, and time-of-day-dependent variation in immunotherapy efficacy. In this more conservative framework, circadian organization may influence the biological conditions under which tumor-reactive T cells expand, persist, or become progressively constrained, but it should not be presented as an established determinant of exhausted-state specification. Thus, biological time represents a testable variable for future longitudinal studies of exhaustion dynamics, rather than a confirmed regulator of exhaustion fate (Wu et al., 2019; Wang et al., 2023; Cervantes-Silva et al., 2022; Wang et al., 2024).

These scheduling questions become more consequential when PD-1 blockade is combined with additional checkpoint inhibitors such as CTLA-4 or LAG-3 blockade. Such regimens can improve response rates in selected settings but also increase the risk of clinically meaningful immune-related adverse events, making efficacy and toxicity inseparable considerations (Tawbi et al., 2022; Bautista et al., 2026; Kyriakidis et al., 2025). In practice, this supports cautious strategies such as staged escalation or time-limited intensification followed by de-escalation, not because a single schedule is universally optimal, but because scheduling itself is a safety-relevant variable that shapes the magnitude, duration, and interpretability of immune activation (Martins et al., 2019; Huang et al., 2017). Overall, vaccines plus PD-1 blockade represent a plausible approach to increase the size and relevance of the responding T cell pool, whereas dual-checkpoint strategies may further amplify responses in selected contexts, but net benefit remains conditional on timing, patient risk, tumor ecology, and careful monitoring of immunologic kinetics and toxicity (Grippin et al., 2025; Khleif and Gupta, 2025).

Engineered cell therapies provide a complementary setting in which signaling history and control layers can be specified more directly. In CAR T systems, synthetic resilience is often pursued by reducing chronic or excessive signaling, preserving the capacity for renewed activation, and maintaining controllability, especially because interventions that improve persistence can also increase toxicity risk (Eyquem et al., 2017; Lynn et al., 2019; Qin and Xu, 2022). In this context, inducible rest is best understood not as a general reversal of exhaustion, but as a programmable interruption of signaling trajectories that may otherwise reinforce constrained differentiation, particularly under tonic or excessive signaling. Drug-regulatable systems and pharmacologic inhibitors can create defined OFF windows, and available data suggest that the duration, timing, and repetition of these intervals influence the extent of functional recovery, proliferative competence, and transcriptional rebalancing after reactivation (Qin and Xu, 2022; Wu et al., 2015; Weber et al., 2021). These observations are more consistent with partial and state-dependent recovery than with uniform identity reset.

This logic extends to context-aware control strategies. Logic gates and fatigue sensors can be designed to restrict activation to permissive conditions or dampen signaling when biochemical indicators of dysfunction accumulate, thereby limiting futile or damaging activation in hostile microenvironments (Teixeira and Fussenegger, 2024; Hernandez-Lopez et al., 2021). Transcription-factor rewiring provides another lever by targeting intrinsic regulatory bottlenecks rather than simply amplifying receptor engagement; for example, augmenting AP-1 activity has improved expansion and function in tonic-signaling CAR models, although such approaches remain bounded by toxicity considerations (Lynn et al., 2019; Chen et al., 2019). Epigenetic editing offers a further prospective strategy to relax stabilizing programs that anchor dysfunctional states, but its most defensible use is likely to be partial and layered, combined with signaling control and explicit safety modules rather than assumed to produce unrestricted reinvigoration (Belk et al., 2022; Ghoneim et al., 2017; Di Stasi et al., 2011).

Taken together, therapeutic control of exhaustion dynamics is best approached not as a single intervention but as a problem of programmable coordination. Across endogenous and engineered settings, the goal is not simply to intensify activation, but to shape when, where, and under what constraints T cells expand, persist, and function. Under this view, the most meaningful endpoints are durable and safety-aware: sustained expansion, preserved reactivation competence, and clinically interpretable antitumor activity achieved without loss of controllability.

## Conclusions, limitations, and future directions

T cell exhaustion is best understood not as a single defect, but as a heterogeneous and constrained organization of antigen-experienced T cell states shaped by chronic stimulation, regulatory architecture, and tumor ecology (Blank et al., 2019). Across tumors, exhausted CD8^+^ T cells occupy phenotypic, transcriptional, epigenetic, metabolic, and spatially distributed configurations that differ in proliferative capacity, effector potential, and responsiveness to therapy. From this perspective, the central therapeutic question is not simply whether exhaustion is present, but which exhausted states are represented, how they are maintained, and which of them remain accessible to productive modulation under treatment (Bassez et al., 2021; Huang et al., 2017). Checkpoint blockade can induce measurable functional improvement, but durable clinical benefit often appears to depend less on uniform reversal of terminal states than on selective expansion of renewal-competent compartments and on whether tumor-imposed boundary conditions permit sustained response (Ghoneim et al., 2017).

At the same time, the field faces important interpretive limitations. Exhaustion is inherently measurement-dependent, and phenotypic, transcriptomic, chromatin, methylation, and spatial definitions capture overlapping but non-identical aspects of T cell state (Blank et al., 2019; Pauken et al., 2016). Most human studies remain constrained by limited temporal resolution, making directionality difficult to infer and increasing the risk of over-interpreting embeddings or trajectory models in the absence of orthogonal validation (Bassez et al., 2021; Bergen et al., 2020). In addition, tissue context, clonal replacement, redistribution between compartments, and interpatient variability can all reshape observed state distributions and complicate attribution of treatment effects to any single mechanism (Yost et al., 2019; DePeaux and Delgoffe, 2021; Thommen et al., 2018). These limitations argue for a stricter distinction between what is directly observed and what is inferred. A further limitation is that current evidence supports circadian effects on T cell antigen responsiveness, dendritic-cell antigen processing, vaccination responses, intratumoral CD8^+^ T cell infiltration and function, and time-of-day-dependent immunotherapy efficacy, but does not yet demonstrate direct circadian specification of exhausted T cell subsets (Wang et al., 2024; Zeng et al., 2024; Cervantes-Silva et al., 2022; Fortier et al., 2011). Future studies should therefore test this question explicitly using longitudinal sampling, time-of-day-controlled tumor models, clonal tracking, and multimodal profiling of TPEX, TEX-int, and TEX-term compartments across circadian phases.

Future progress will depend on replacing broad claims of reinvigoration with more precise and testable frameworks. This includes standardized state definitions, longitudinal and multimodal study designs, and clinically feasible metrics that capture reservoir status, state composition, clonal continuity, and spatial organization before and during therapy (Siddiqui et al., 2019; Saelens et al., 2019). It also requires treating timing, sequencing, and microenvironmental context as primary therapeutic variables rather than secondary considerations, because the consequences of inhibitory relief are likely to depend on when it is applied, which exhausted compartments remain available, and whether metabolic, spatial, and epigenetic constraints can be partially relaxed without compromising fitness or safety (Binnewies et al., 2018; Khan et al., 2019; Schietinger et al., 2016). In engineered systems, inducible rest, context-aware gating, transcriptional rewiring, and integrated safety modules offer a tractable framework for testing how persistence, controllability, and toxicity can be balanced more rationally (Abreu et al., 2026).

Overall, the most credible view of exhaustion is one of constrained dynamics rather than binary failure. Therapeutic progress is therefore unlikely to come from a single intervention that reverses exhaustion wholesale, but from strategies that more precisely measure, preserve, and redirect T cell state distributions within the regulatory, metabolic, and spatial limits imposed by tumors. In that sense, the future of the field lies not in asking whether exhausted T cells can be rescued in general, but in defining which exhausted states can still be productively controlled, when, and under what conditions.
